# Identification and Validation of a New Functional Gene *TSC22D3* for hBMSCs Osteogenesis

**DOI:** 10.1155/sci/1047964

**Published:** 2025-11-11

**Authors:** Shuhong Zhang, Xiao Li, Zhanping Yang, Jialong Li, Gang Liu, Yongkun Sun, Huigen Feng, Xianwei Wang

**Affiliations:** ^1^Henan Key Laboratory of Medical Tissue Regeneration, Xinxiang Medical University, Xinxiang, Henan, China; ^2^Henan Key Laboratory of Medical Tissue Regeneration, Xinxiang Medical University, The Third Affiliated Hospital of Xinxiang Medical University, Xinxiang, Henan, China; ^3^School of Life Sciences and Biotechnology, Sanquan College of Xinxiang Medical University, Xinxiang, Henan, China

**Keywords:** hBMSCs, osteogenic differentiation, *TSC22D3*

## Abstract

**Background:**

Osteogenic differentiation is a crucial process in which bone marrow mesenchymal stem cells (BMSCs) differentiate into osteoblasts, involving the regulation of multiple genes and signaling pathways. The *TSC22D3* gene plays an important role in various biological processes (BPs), but its specific function in osteogenic differentiation remains unclear. This study aims to explore the regulatory role of the *TSC22D3* gene in osteogenic differentiation and its molecular mechanisms.

**Methods:**

By analyzing microarray datasets (GSE12266, GSE18043, and GSE80614), the limma package was used to screen for differentially expressed genes (DEGs). Combined with Gene Ontology (GO) and Kyoto Encyclopedia of Genes and Genomes (KEGG) enrichment analyses, key genes and signaling pathways related to osteogenic differentiation were identified. Further, through protein–protein interaction (PPI) network analysis and the Finding Regulatory Elements by Differential Expression and Network-Based Statistical Analysis (FRIEND) method, *TSC22D3* was screened out as a core hub gene. For experimental validation, the bioinformatics analysis results were intersected with the transcriptome sequencing data from our research group to further confirm the core molecules. Lentivirus-mediated interference technology was used to downregulate and overexpress *TSC22D3* expression, and the impact of *TSC22D3* on osteogenic differentiation was assessed through RT-qPCR, Western blotting, alkaline phosphatase (ALP) staining, phalloidin staining, and calcium deposition assays.

**Results:**

*TSC22D3* is significantly upregulated during osteogenic differentiation; its downregulation can lead to reduced expression of osteogenic differentiation marker genes (such as runt-related transcription factor 2 [Runx2], osterix [OSX], osteocalcin [OCN], and osteopontin [OPN]), as well as a significant decrease in ALP activity and calcium deposition. GO and KEGG analyses indicate that *TSC22D3* is closely associated with pathways including the cell cycle, cytoskeleton, and WNT signaling. Furthermore, Gene Set Enrichment Analysis (GSEA) analysis has further revealed the potential regulatory mechanism of *TSC22D3* in osteogenic differentiation. Rescue experiments have confirmed that *TSC22D3* can promote the osteogenic differentiation of BMSCs and induce the rearrangement of cytoskeletal structure.

**Conclusion:**

This study reveals that *TSC22D3* is essential for osteogenic differentiation. Its upregulation promotes osteogenic marker expression, ALP activity, and calcium deposition, while its downregulation inhibits these processes. *TSC22D3* affects cytoskeletal rearrangement during osteogenesis.

## 1. Introduction

Skeletal pathologies, such as osteoporosis and osteoarthritis, involve complex processes that affect bone formation, remodeling, and function, ultimately leading to severe functional impairment [1]. Bone marrow mesenchymal stem cells (BMSCs) can self-renew and differentiate into osteoblasts, chondrocytes, and adipocytes, making them valuable for clinical applications [2]. BMSCs play a crucial role in bone repair through osteogenic differentiation, which is regulated by complex gene networks and signaling pathways [3]. In vivo studies have shown that stem cell fate and differentiation are influenced by factors such as the matrix microenvironment, scaffold composition, and cytokines [4]. Understanding the molecular mechanisms controlling osteogenic differentiation is essential for optimizing bone scaffold transplantation and improving bone injury repair [5]. While high-throughput sequencing has helped deepen our understanding of osteogenic differentiation, there remains a limitation in integrating data to identify key regulatory genes, and this hinders our comprehensive understanding of the relevant molecular mechanisms.

The *TSC22D3* gene encodes a glucocorticoid-induced leucine zipper (GILZ), which plays an important role in the anti-inflammatory and immunosuppressive responses [6]. This protein can inhibit proinflammatory molecules and is involved in various biological processes (BPs) such as immune regulation and cell proliferation [7]. Recent research has expanded our understanding of *TSC22D3* and explored its role in different diseases and conditions. For example, it has been identified as an immune-related prognostic biomarker in acute myeloid leukemia (AML) [8], and its expression is associated with patient prognosis and tumor microenvironment [9]. In addition, the study also demonstrated the involvement of *TSC22D3* in stress response, particularly in the context of cancer. The study suggests that *TSC22D3* may influence cell fate determination by regulating transcription factors or epigenetic modifications [10]. These findings highlight the different roles of *TSC22D3* in health and disease, but its specific mechanism in BMSCs osteogenic differentiation still needs further investigation. Additionally, current osteogenic differentiation-related gene screening often relies on single datasets, which may introduce technical bias, and there is a need for multidataset joint analysis to improve the reliability of the results.

This study identifies key osteogenic differentiation genes through integrated analysis of microarray datasets (GSE12266, GSE18043, and GSE80614) and our transcriptome sequencing data. Using differentially expressed genes (DEGs) screening, enrichment analysis, and a protein–protein interaction (PPI) networks, we systematically mapped BPs and identified hub genes. Functional validation of *TSC22D3* was performed through lentiviral interference, RT-qPCR, Western blotting, and osteogenic assays in BMSCs. These research findings deepen the understanding of osteogenic mechanisms and provide potential therapeutic targets for bone diseases.

## 2. Materials and Methods

### 2.1. Data Processing and Analysis

#### 2.1.1. DEG Screening and Gene Enrichment Analysis

DEGs were identified from microarray datasets (GSE12266, GSE18043, and GSE80614) using the limma package (Supporting Information File 1). To address false discovery rates in multiple testing, adjusted *p*-values were applied. DEGs distinguishing osteogenic differentiation samples from normal controls were selected using stringent thresholds (adjusted *p* < 0.05 and log2 fold-change ≥0.5). Genes showing consistent upregulation or downregulation in at least two datasets were prioritized (Supporting Information File 2). Gene Ontology (GO) and Kyoto Encyclopedia of Genes and Genomes (KEGG) enrichment analyses were performed on these DEGs using the clusterProfiler package in R.

#### 2.1.2. Identification and Analysis of Hub Genes in Osteogenic Differentiation

Hub genes and regulatory networks were identified through integrated bioinformatics approaches. First, the Finding Regulatory Elements by Differential Expression and Network-Based Statistical Analysis (FRIEND) method was applied using the GOSemSim package in R to select the top 50 candidate genes based on GO enrichment results (Supporting Information File 3). Concurrently, a PPI network was constructed using DEGs via the STRING database. The cytoHubba plugin in Cytoscape was used to identify the top 50 PPI network genes. Intersection of these two gene lists yielded final hub genes, with high-scoring candidates selected for experimental validation (Supporting Information File 4). Subsequent analyses of the hub genes included correlation analysis, GO, KEGG, and Gene Set Enrichment Analysis (GSEA) (Supporting Information File 5).

The results of the aforementioned bioinformatics analysis were intersected with the transcriptomic sequencing data from our previous experiments on osteogenic differentiation induction in rat BMSCs, to further identify the core molecules closely associated with osteogenic differentiation.

### 2.2. Main Materials and Reagents

Human BMSCs (hBMSCs) were obtained from Saiye Biotechnology Co., Ltd. (product number: HUXMA-01001). Lentivirus-mediated interference plasmid for downregulation of *TSC22D3* was custom-synthesized by Anshengda Biotechnology Co., Ltd. (order number: 80-839735734). Osteogenic induction medium (OIM), consisting of the following components: Dulbecco's Modified Eagle Medium (DMEM), 10% fetal bovine serum (FBS), 10 mM β-glycerophosphate, 50 μg/mL L-ascorbic acid, 0.1 μmol/L dexamethasone, and 1% double antibiotic. Alkaline phosphatase (ALP) assay kit (Nanjing Jiancheng Bioengineering Institute, product number: A059-2); calcium assay kit (Nanjing Jiancheng Bioengineering Institute, product number: C004-2-1); *TSC22D3* antibody (Proteintech, product number: 12352-1-AP).

### 2.3. Main Experimental Methods

#### 2.3.1. Cell Culture and Osteogenic Induction

hBMSCs were maintained in minimum essential medium alpha (α-MEM) supplemented with 10% FBS at 37°C in a 5% CO_2_ humidified incubator. Cells at passages 3–5 were used for experiments. Osteogenic differentiation was induced by culturing hBMSCs in OIM medium for the required experimental duration.

#### 2.3.2. Transcriptome Sequencing Analysis

The research team previously extracted BMSCs from rats and conducted primary culture. Cells from the P4 generation were selected and induced to undergo osteogenic differentiation in OIM for 7 days. RNA was then extracted and sent to a company (Shanghai Paisenuo Biotechnology Co., Ltd., contract number: PN20241127035) for transcriptome sequencing. The samples were categorized into two groups: a control group and an osteogenic differentiation group (OS) (Supporting Information File 6). Subsequently, the sequencing results underwent comprehensive bioinformatics analysis.

#### 2.3.3. Lentiviral Interference

Lentiviral packaging was performed in 293T cells using the target shRNA, psPAX2, and Pmg2G plasmids at a ratio of 6:4:2. After 48–60 h of transfection, the supernatant from the 293T cells was collected. Each well containing hBMSCs was supplemented with 1 mL of the 293T supernatant, followed by the addition of 1 mL of OIM medium to each well. Finally, approximately 8 μg/mL polybrene solution was added to each well. The wells were gently mixed, and 4–6 h after transfection, the medium was replaced with fresh OIM.

#### 2.3.4. RT-qPCR and Western Blotting

In this study, the expression of hub genes and osteogenic marker genes was identified by RT-qPCR and Western blotting. The primer sequences used in this experiment were listed in Table 1. The experimental results were plotted and statistically analyzed using Primer 8.0 software.

#### 2.3.5. Osteogenic Differentiation Related Assays

ALP staining was assessed using the BCIP/NBT ALP chromogenic reagent kit, and ALP activity was measured using an ALP assay kit. Calcium deposition in hBMSCs was evaluated through Alizarin Red (ARS) staining and a calcium assay kit. The experimental results were analyzed for grayscale using Image Proplus 6.0 software.

#### 2.3.6. Cytoskeleton Staining

Use a phalloidin kit to stain the cytoskeleton. First, place the target adherent cells (cultured to 50%–80% confluency) on a coverslip. After aspirating the culture medium, fix the cells with precooled 4% paraformaldehyde at room temperature for 10–15 min. Next, permeabilize the cells with PBS containing 0.1%−0.5% Triton X-100 at room temperature for 5–10 min, followed by blocking with PBS containing 1%–5% bovine serum albumin (BSA) at room temperature for 30 min. Subsequently, dilute the fluorescently labeled phalloidin in proportion, cover the cells with it, and incubate in the dark at room temperature for 20–30 min. Then, add DAPI staining solution and incubate in the dark at room temperature for 5–10 min. After each incubation step, wash the cells with PBS three times (5 min each time). Finally, mount the sample with antifluorescence quenching mounting medium and perform scanning imaging.

#### 2.3.7. Statistics

Statistically significant differences between means were determined using the GraphPad Prism 8.0 software. Unless stated otherwise, an unpaired Welch's *t* test was performed for the comparison of two groups. Results were considered significant at *p* < 0.05.

## 3. Results

### 3.1. Identification and Analysis of DEGs During Osteogenic Differentiation Process

**Figure 1:**
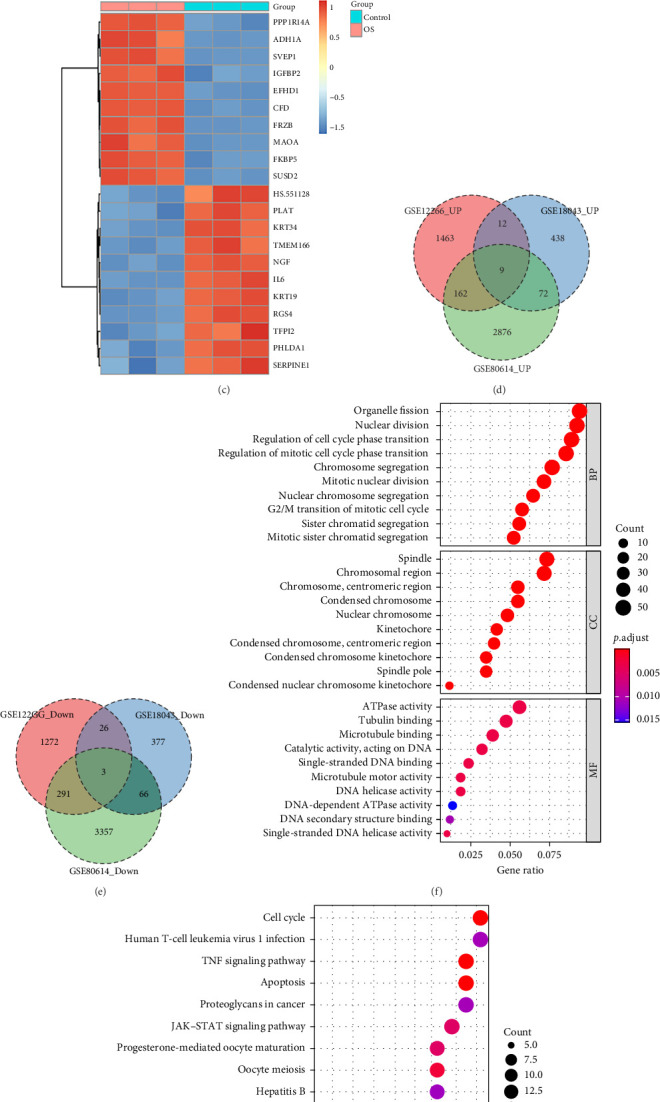
DEGs Analysis. Heatmaps (A, B, and C) display the top 20 most differentially expressed genes across three datasets (GSE80614, GSE12266, and GSE18043). A color gradient from red (high expression) to blue (low expression) represents gene expression levels. (D) Overlapping upregulated genes identified from differential expression analysis. (E) Overlapping downregulated genes from the same analysis. (F) GO enrichment analysis, including biological processes (BPs), molecular functions (MFs), and cellular components (CCs). (G) KEGG pathway enrichment analysis. The *y*-axis represents GO terms or KEGG pathways, while the *x*-axis indicates the gene ratio. The color gradient (blue to red) reflects the enrichment significance, and the dot size corresponds to the number of genes associated with each pathway.

Heatmaps (Figure 1A–C) were employed to highlight the most significant genes among all DEGs within the OS in comparison to the control group. Conduct an intersection analysis of DEG within the three datasets. Intersection analysis identified nine upregulated genes (Figure 1D) and three downregulated genes (Figure 1E). Subsequently, prioritize genes that exhibit concurrent upregulation or downregulation in at least two of the datasets for further in-depth analysis.

We performed GO analysis on DEGs to identify key functional aspects of genes in osteogenic differentiation (Figure 1F). Key findings included BPs like organelle fission, nuclear division, and cell-cycle phase transition regulation. Cellular components (CCs) such as the spindle and chromosomal regions were also highlighted. Molecular functions (MFs) linked to osteogenic differentiation included ATPase activity and tubulin/microtubule binding. We performed KEGG pathway analysis to identify signaling pathways involved in the osteogenic differentiation of hBMSCs, revealing 294 significant pathways (*p* < 0.05). Key pathways included those related to the cell cycle, human T-cell leukemia virus 1 infection, TNF signaling, apoptosis, and proteoglycans in cancer (Figure 1G). These results enhance our understanding of the molecular mechanisms underlying osteogenic differentiation in hBMSCs.

### 3.2. Screening and Bioinformatics Analysis of Hub Gene *TSC22D3*

**Figure 2:**
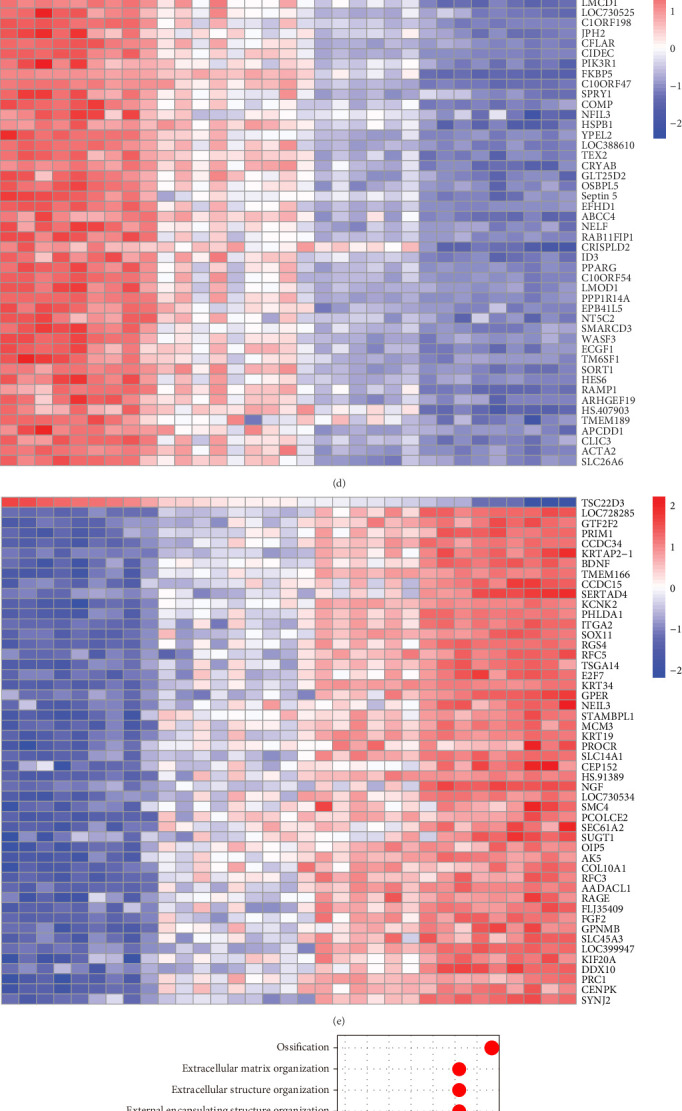
Identification and bioinformatic analysis of hub genes. (A) Functional similarity distributions are visualized using boxplots, which provide a concise summary of the data. The boxes represent the interquartile range (IQR), encompassing the middle 50% of the functional similarities, with the upper and lower boundaries indicating the 75th and 25th percentiles, respectively. The horizontal lines within the boxes represent the mean values of the functional similarities. A dashed line is included to indicate the cutoff value. (B) The top 50 key genes identified by the Betweenness algorithm in cytoHubba. Red nodes represent genes with higher scores, while yellow nodes denote genes with relatively lower scores. (C) Venn diagram showing the overlap between the top 50 genes identified by cytoHubba and the top 50 genes identified using the FRIENDS method. (D) Heatmap displaying genes positively correlated with *TSC22D3*. Red indicates upregulation, while blue indicates downregulation. (E) Heatmap displaying genes negatively correlated with *TSC22D3*. (F) GO analysis of the *TSC22D3* gene. (G) KEGG pathway analysis of the *TSC22D3* gene. (H) Results of GSEA based on Reactome. The abscissa represents the enrichment score (ES); an ES > 0 indicates a positive correlation between the gene and the pathway, while an ES < 0 indicates a negative correlation.

To identify hub genes, we selected the top 50 candidate genes using the FRIENDS method (Figure 2A), including *TROAP*, *FANCA*, *and TSC22D3*, and the top 50 key genes from PPI analysis (Figure 2B), such as *IL6*, *BRCA1*, *CCNB1*, *and VEGFA*. Intersection analysis of these genes revealed four hub genes: *TSC22D3*, *BIRC5*, *GMNN*, *and BRCA1* (Figure 2C). *TSC22D3*, with the highest FRIENDS score, was identified as the primary hub gene. Correlation analysis showed genes positively and negatively associated with *TSC22D3* (Figure 2D, E), indicating functional links.

GO analysis of *TSC22D3* highlighted its role in osteogenic differentiation (Figure 2F), while pathway analysis linked it to human papillomavirus infection, cytoskeleton regulation, and WNT signaling (Figure 2G). GSEA using reactome pathways (Figure 2H) further revealed subtle yet significant gene expression changes, aiding in understanding upstream and downstream regulatory mechanisms.

### 3.3. Experimental Verification of *TSC22D3* Expression and Interference

**Figure 3:**
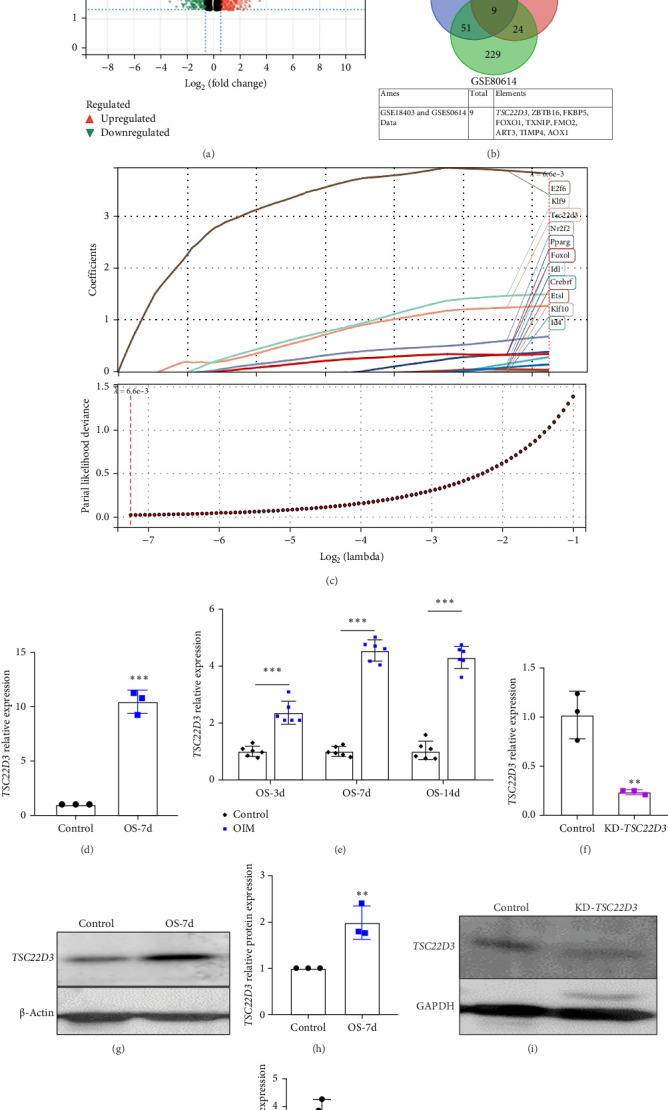
Detection of *TSC22D3* expression in osteosarcoma cells and knockdown validation. (A) Volcano plot of sequencing results from our research group. (B) Overlapping DEGs between our sequencing data and datasets GSE18403/GSE80614. (C) LASSO regression analysis of transcriptomic sequencing data from our research group. (D) Expression levels of the *TSC22D3* gene in our sequencing dataset. (E) RT-qPCR analysis revealed upregulated *TSC22D3* mRNA expression in osteogenically induced cells at 3, 7, and 14 days compared to controls. (F) Validation of *TSC22D3* knockdown efficiency by RT-qPCR. (G) WB demonstrating elevated *TSC22D3* protein expression in OS cells. (H) Grayscale quantification statistical analysis of WB results. (I) WB validation of lentiviral interference efficiency. (J) Grayscale quantification confirmed significantly reduced *TSC22D3* expression in KD-*TSC22D3* cells versus controls (*p* < 0.05). *⁣*^*∗∗*^ denotes *p* < 0.01 (highly significant) and *⁣*^*∗∗∗*^ denotes *p* < 0.001 (extremely significant).

In previous studies, our research group has completed transcriptome sequencing of a rat BMSC osteogenic induction model. The volcano plot (Figure 3A) and bar chart (Figure 3D) demonstrate that *TSC22D3* showed significantly higher expression in osteogenically induced cells compared to the control group in these transcriptomic sequencing results. The Venn diagram (Figure 3B) reveals the intersection of upregulated DEGs between our transcriptome sequencing data and two datasets (GSE18403 and GSE80614), identifying nine coupregulated genes, including *TSC22D3*. LASSO analysis of the sequencing results (Figure 3C) further indicated that *TSC22D3* ranked prominently among the top regression coefficients (top 3, coefficient = 1.28). These experimental findings collectively suggest that the *TSC22D3* gene may play a significant role in osteogenic differentiation processes.

To validate the findings from our bioinformatics analysis, we examined both mRNA and protein expression levels of *TSC22D3* in hBMSCs. The RT-qPCR results demonstrated a significant upregulation of *TSC22D3* expression in cell samples undergoing osteogenic differentiation at 3, 7, and 14 days compared to the control group (day 0) (Figure 3E). Western blotting analysis (Figure 3G, H) further confirmed that *TSC22D3* protein expression increased following osteogenic induction.

In this study, we utilized lentiviral transduction to knockdown (KD) *TSC22D3* expression in hBMSCs. To validate the KD efficiency, we performed comprehensive assessments using both RT-qPCR and Western blotting analyses, as shown in Figure 3F, I, J.

### 3.4. The *TSC22D3* Gene Plays a Crucial Role in the Osteogenic Differentiation of hBMSCs

**Figure 4:**
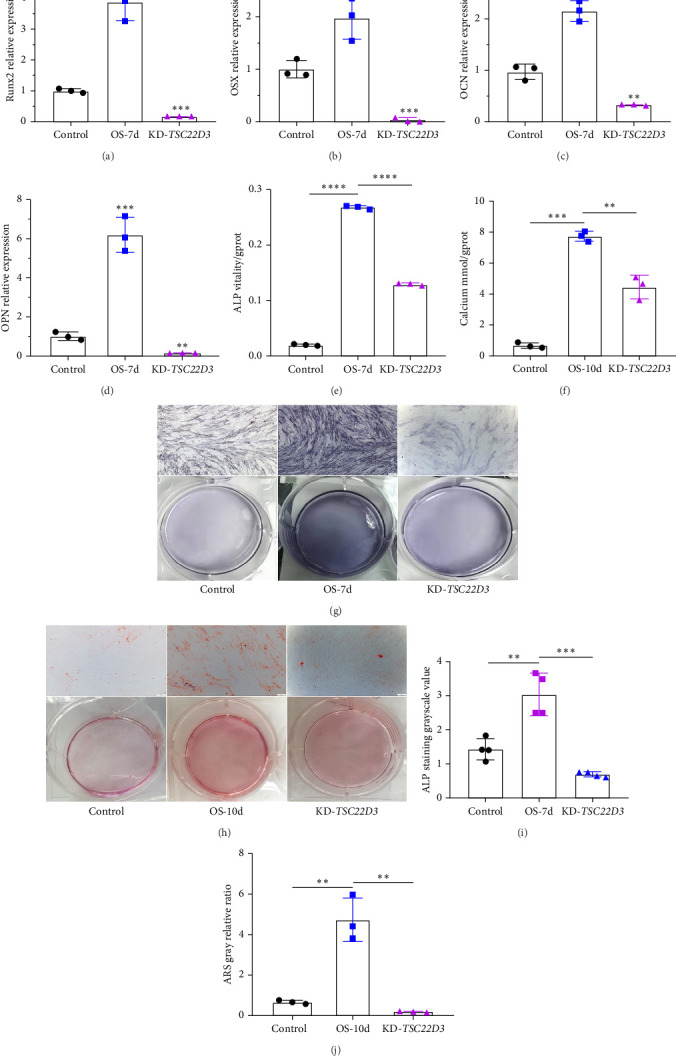
The correlation of *TSC22D3* with osteogenic differentiation was assessed. A, B, C, and D used the RT-qPCR method to detect the expression of osteogenic marker genes. The groups were control, OS-7d, and KD-*TSC22D3*. (E) Compared to the OS-7d group, ALP activity decreased in KD-*TSC22D3* cells. (F) Calcium content was measured using a calcium deposition assay kit, and compared to the OS-7d group, calcium deposition decreased in KD-*TSC22D3* cells. (G) ALP staining. (I) ALP staining statistical analysis. (H) ARS staining. (J)ARS staining statistical analysis. Compared with the OS-7d group, the ALP content and calcium deposition in hBMSCs decreased after downregulation of the *TSC22D3* gene, Scale bar: 100 μm. Different symbols represent data from different groups. Circles indicate the control group, squares represent the OS group, and triangles represent the KD-TSC22D3 group. *⁣*^*∗*^*p* < 0.05, *⁣*^*∗∗*^*p* < 0.01, *⁣*^*∗∗∗*^*p* < 0.001, *⁣*^*∗∗∗∗*^*p* < 0.0001.

After downregulating the expression of *TSC22D3* gene, we evaluated the expression levels of key osteogenic marker genes runt-related transcription factor 2 (Runx2), osterix (OSX), osteocalcin (OCN), and osteopontin (OPN) using RT-qPCR technology, and detected ALP activity and calcium deposition. Contrast to the control group, the KD of *TSC22D3* (KD-*TSC22D3*) resulted in a substantial reduction in the expression of osteogenic marker genes (Figure 4A–D), a concomitant decrease in ALP activity (Figure 4E), and a discernible diminishment in calcium deposition (Figure 4F). Collectively, these experimental findings lend credence to the hypothesis that the *TSC22D3* gene exerts a regulatory influence over the osteogenic differentiation process in hBMSCs.

ALP and ARS staining assays were carried out on cellular specimens derived from the control group, the OS-7d group, and the KD-*TSC22D3* group, as depicted in Figure 4G–J. Notably, within the KD-*TSC22D3* cell population, a conspicuous reduction in both ALP content and calcium deposition was observed. This compelling observation implies a pivotal role of the *TSC22D3* gene in modulating the osteogenic differentiation process of hBMSCs, thereby underscoring the need for further experimental validation to elucidate the precise underlying mechanisms.

### 3.5. Overexpressing *TSC22D3* Promotes hBMSCs Osteogenic Differentiation and Cytoskeleton Rearrangement

**Figure 5:**
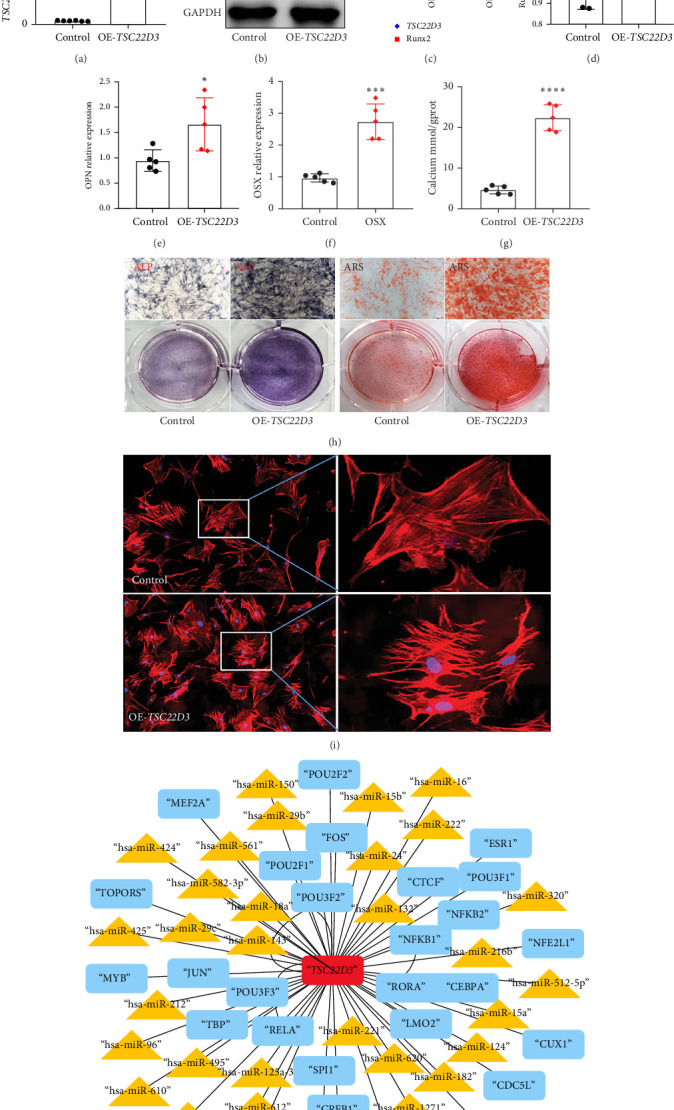
Overexpression of *TSC22D3* gene promotes osteogenic differentiation and cytoskeletal rearrangement of hBMSCs. (A) Efficiency of *TSC22D3* overexpression identified by RT-qPCR. (B) Western blot analysis for detecting the efficiency of *TSC22D3* overexpression and the expression of Runx2. (C) Gray value analysis of Western blot results. (D, E, and F) Expression of osteogenic marker genes detected by RT-qPCR. (G) Calcium content measured using a calcium deposition assay kit. (H) ALP staining and ARS staining. Scale bar: 100 μm. (I) Cytoskeletal system stained with phalloidin. (J) Bioinformatics analysis predicting transcriptional regulators and miRNAs of the *TSC22D3* gene. Rectangles represent transcription factors and triangles represent miRNAs. Circles indicate the control group data. *⁣*^*∗*^*p* < 0.05, *⁣*^*∗∗*^*p* < 0.01, *⁣*^*∗∗∗*^*p* < 0.001, *⁣*^*∗∗∗∗*^*p* < 0.0001.

To further verify the correlation between *TSC22D3* and the osteogenic differentiation of BMSCs, we overexpressed *TSC22D3* in hBMSCs via lentiviral packaging and transfection, and verified the indicators of osteogenic differentiation. First, RT-qPCR and Western blot were used to detect the efficiency of *TSC22D3* overexpression (OE). Compared with the control group, the mRNA and protein levels of the target gene *TSC22D3* in the OE group (OE-*TSC22D3*) were significantly upregulated (Figure 5A–C). Meanwhile, it was detected that after OE-*TSC22D3*, the expression of Runx2 protein was upregulated (Figure 5B,C). We evaluated the expression levels of key osteogenic marker genes Runx2, OPN, and OSX using RT-qPCR technology, and detected calcium deposition. Compared with the control group, OE-*TSC22D3* resulted in an increase in the expression of osteogenic marker genes (Figure 5D–F), and an increase in calcium deposition (Figure 5G). In ALP and ARS staining assays, enhanced ALP and ARS staining was observed in the OE-*TSC22D3* group compared with the control group (Figure 5H). All these experimental results suggest that *TSC22D3* can promote the osteogenic differentiation of hBMSCs.

Since previous bioinformatics analysis showed that *TSC22D3* is closely related to the cytoskeletal system (Figure 2G, H), we used phalloidin to stain the cytoskeleton of hBMSCs with OE-*TSC22D3*. The results, as shown in Figure 5I, revealed structural rearrangement of the cytoskeleton, with a visible increase in the proportion of clustered and longitudinally arranged cytoskeletal structures. This result further indicates that *TSC22D3* may be involved in the osteogenic differentiation process by altering the cytoskeletal structure. In addition, through bioinformatics analysis, we screened potential transcription factors and miRNAs that may regulate *TSC22D3* (Figure 5J), which further suggests its possible molecular mechanism.

## 4. Discussion

This study systematically explored the regulatory role of *TSC22D3* gene in the osteogenic differentiation of hBMSCs by integrating bioinformatics analysis and experimental verification. The research results revealed the potential mechanism of *TSC22D3* as a key hub gene in osteogenic differentiation, and its importance was verified through functional experiments, providing a new perspective for a deeper understanding of the molecular regulatory network of osteogenic differentiation.

Chronic diseases or injuries that damage bone tissue can disrupt daily life and complicate treatment. Repair often requires in vitro scaffolds or composite structures with seed cells [11]. The use of hBMSCs in tissue-engineered bone repair has gained attention [12]. While stem cells hold promise for tissue repair, their regenerative potential is influenced by complex molecular networks, and the underlying mechanism remains unclear.

Research on stem cell differentiation and regeneration now focuses on understanding the cells and signaling molecules in the local matrix microenvironment [13]. Signaling regulation is key to osteogenic differentiation, but the exact mechanisms behind osteogenesis, tissue regeneration, and repair remain unclear, limiting clinical applications. BMSCs are typically induced to become osteoblasts under certain conditions. Studies have highlighted the role of key signaling pathways, such as TGF-β/BMP [14], WNT [15], and FGF [16], in regulating BMSCs differentiation and lineage commitment [17]. The biological modules and core genes identified through experimental validation may ultimately serve as biomarkers for the detection and treatment of bone injury [18].

This study identified the pivotal gene *TSC22D3*, which is significantly upregulated during osteogenic differentiation, through a joint analysis of multiple datasets. Experimental validation showed that the expression of *TSC22D3* was significantly increased at day 3, 7, and 14 of osteogenic induction. KD-*TSC22D3* significantly inhibited the expression of osteogenic marker genes (such as Runx2, OSX, OCN, and OPN), and decreased aALP activity and calcium deposition levels. OE-*TSC22D3*; however, promotes osteogenic differentiation. This finding provides the first evidence of the positive regulatory role of *TSC22D3* in osteogenic differentiation of hBMSCs, filling the gap in the study of this gene in bone metabolism research.

Our GO and KEGG analyses revealed several interconnected BPs related to BMSCs osteogenic differentiation, including organelle fission, nuclear division, and cell-cycle phase regulation. Notably, signal regulation governing cell cycle phase transitions has implications in various biological phenomena such as obesity, hyperinsulinemia, hormonal regulation, the local microenvironment, and cellular aging [19–21]. Our biological analysis identified fluid shear stress as a factor influencing osteogenic differentiation. Studies indicate that fluid shear stress regulates vascular tension and blood pressure via endothelial adrenomedullin signaling [22, 23]. This discovery indirectly suggests that there may be some network like regulatory relationships between abnormal regulation of osteogenic differentiation and the occurrence and development of a series of diseases in the body, which can be further explored.

GO and KEGG analyses of the hub gene *TSC22D3* showed that this gene is closely related to osteogenic differentiation regulation, bone mineralization, cytoskeleton regulation, WNT signaling pathway, and TGF-β signaling pathway. In addition, LASSO regression analysis and GSEA results suggest that *TSC22D3* may exert its effects through synergistic or independent pathways, complementing the regulatory network of classical osteogenic transcription factors such as Runx2 and OSX recently discovered [24]. The experimental results also confirmed that *TSC22D3* can induce cytoskeletal rearrangement. The above experimental results further support the important role of *TSC22D3* in the regulation of osteogenic differentiation. However, the specific regulatory mechanism of *TSC22D3* within these pathway remains to be further elucidated.

*TSC22D3* is a protein encoding member of the TSC-22/DIP/Bun family, as its role in certain diseases has also received attention in recent years. Especially in glucocorticoid signaling, *TSC22D3* is a key regulatory molecule [25]. Corticosteroids play an important role in treating certain bone diseases, such as rheumatoid arthritis, but long-term or high-dose use of glucocorticoids can inhibit bone formation, increase the risk of osteoporosis, and fractures. *TSC22D3* is induced to express under the action of glucocorticoids and may indirectly affect bone metabolism by regulating inflammatory responses, cell proliferation, and other processes, especially in regulating bone diseases caused by glucocorticoids such as osteoporosis [26]. Studies have shown that upregulation of *TSC22D3* can alleviate glucocorticoid related bone damage by improving the function of osteoblasts and reducing bone resorption [27]. Researchers may explore its potential as a drug target. In patients with long-term use of glucocorticoids, the expression of *TSC22D3* may become an indicator for evaluating the risk of osteoporosis, helping doctors assess bone health status and take corresponding intervention measures.

In addition, the downregulation and loss of *TSC22D3* in vitro and in vivo contributes to the aging process of macrophages and peripheral blood leukocytes [28]. The decrease in *TSC22D3* mRNA and protein levels is associated with an increase in inflammation severity in systemic lupus erythematosus (SLE), ulcerative colitis, psoriasis, and other autoimmune inflammatory diseases [29]. It is worth noting that *TSC22D3* exhibits enhanced expression during T cell transformation and has clinical relevance in tumor staging [30]. In the bioinformatics analysis of cervical cancer-related genes, *TSC22D3* has significant features in the cell proliferation signaling system [31]. *TSC22D3* is highly expressed in the brain tissue of morphine tolerant mice, activating the iron death pathway, enhancing cell apoptosis and promoting brain cell inflammatory response [32]. The expression of *TSC22D3* in liver biopsy samples of patients with liver fibrosis is negatively correlated with the expression of CCL2 [33].

In conclusion, the *TSC22D3* gene plays a crucial role in a variety of physiological and pathological processes, and it has attracted increasing attention as a potential therapeutic target. Although the *TSC22D3* gene is associated with multiple diseases, its connection to osteogenesis has been rarely studied. Through analysis and experimental verification, this study confirmed the association between *TSC22D3* and osteogenic differentiation, laying a foundation for in-depth exploration of the molecular mechanism and functional role of this gene in osteogenic differentiation.

This section discusses the strengths and weaknesses of the methodology used in the study. The research applied a thorough bioinformatics approach (e.g., limma package for gene screening and STRING database for PPI network construction), alongside experimental validation (e.g., RT-qPCR, Western blot, and phenotype detection), to ensure result reliability. However, there are some limitations: the specific downstream targets of *TSC22D3* and their relationship with signaling pathways remain unclear, requiring further exploration using techniques like ChIP-seq or dual luciferase reporter assays.

Future research on *TSC22D3* can focus on: (1) exploring its regulatory network and interactions with osteogenic pathways like WNT, TGF-β, and BMP; (2) creating a conditional knockout mouse model to study its role in bone formation and regeneration; (3) investigating its epigenetic regulation, such as methylation or noncoding RNA; (4) developing molecular interventions targeting *TSC22D3* for potential applications in osteoporosis or bone defect treatment.

## 5. Conclusions

The identified gene *TSC22D3* is a key regulator in osteogenic differentiation, which provides a novel biological insight in osteogenic differentiation of BMSCs. This study reveals that *TSC22D3* is essential for osteogenic differentiation. Its upregulation promotes osteogenic marker expression, ALP activity, and calcium deposition, while its downregulation inhibits these processes. *TSC22D3* affects cytoskeletal rearrangement during osteogenesis. The research findings of this article provide a potential therapeutic target for bone injury repair and in vitro bone tissue engineering.

## Figures and Tables

**Table 1:** Primer sequences.

Gene	F (5′ to 3′)	R (5′ to 3′)
*TSC22D3*	GAAGGAGCAGATCCGAGAGC	GGCTCAGACAGGACTGGAAC
Osteopontin (OPN)	AGCAGCTTTACAACAAATACCCAG	TTACTTGGAAGGGTCTGTGGG
Osteocalcin (OCN)	CACACTCCTCGCCCTATTG	GGTCTCTTCACTACCTCGCT
Runt-related transcription factor 2 (RUNX2)	TTCCAGAATGCTTCCGCCAT	AACTGCTGTGGCTTCCATCA
Osterix (OSX)	TCTGCGGGACTCAACAACTC	TAGCATAGCCTGAGGTGGGT
GAPDH	GAAAGCCTGCCGGTGACTAA	GCATCACCCGGAGGAGAAAT

## Data Availability

The datasets used or analyzed during the current study are available from the corresponding author upon reasonable request.

## Supporting Information

Supporting Information 1 Supporting Information File 1: Contains the differential expression gene analysis results for datasets GSE12266, GSE18043, and GSE80614. Supporting Information File 2: Contains the intersection results of differentially expressed genes across datasets GSE12266, GSE18043, and GSE80614. Supporting Information File 3: Contains hub gene identification results via FRIENDS analysis. Supporting Information File 4: Contains the intersection results of hub genes. Supporting Information File 5: Contains the single-gene enrichment analysis results for *TSC22D3*. Supporting Information File 6: Contains the original high-throughput sequencing data of BMSCs induced by osteogenic differentiation and the LASSO analysis results for the *TSC22D3* gene.
